# Chaperone expression profiles correlate with distinct physiological states of *Plasmodium falciparum *in malaria patients

**DOI:** 10.1186/1475-2875-9-236

**Published:** 2010-08-19

**Authors:** Rani Pallavi, Pragyan Acharya, Syama Chandran, Johanna P Daily, Utpal Tatu

**Affiliations:** 1 Department of Biochemistry, Indian Institute of Science, Bangalore, 560012, Karnataka, India; 2 Department of Medicine, Albert Einstein College of Medicine, 1301 Morris Park Avenue, Bronx, New York 10461, USA

## Abstract

**Background:**

Molecular chaperones have been shown to be important in the growth of the malaria parasite *Plasmodium falciparum *and inhibition of chaperone function by pharmacological agents has been shown to abrogate parasite growth. A recent study has demonstrated that clinical isolates of the parasite have distinct physiological states, one of which resembles environmental stress response showing up-regulation of specific molecular chaperones.

**Methods:**

Chaperone networks operational in the distinct physiological clusters in clinical malaria parasites were constructed using cytoscape by utilizing their clinical expression profiles.

**Results:**

Molecular chaperones show distinct profiles in the previously defined physiologically distinct states. Further, expression profiles of the chaperones from different cellular compartments correlate with specific patient clusters. While cluster 1 parasites, representing a starvation response, show up-regulation of organellar chaperones, cluster 2 parasites, which resemble active growth based on glycolysis, show up-regulation of cytoplasmic chaperones. Interestingly, cytoplasmic Hsp90 and its co-chaperones, previously implicated as drug targets in malaria, cluster in the same group. Detailed analysis of chaperone expression in the patient cluster 2 reveals up-regulation of the entire Hsp90-dependent pro-survival circuitries. In addition, cluster 2 also shows up-regulation of *Plasmodium *export element (PEXEL)-containing Hsp40s thought to have regulatory and host remodeling roles in the infected erythrocyte.

**Conclusion:**

In all, this study demonstrates an intimate involvement of parasite-encoded chaperones, PfHsp90 in particular, in defining pathogenesis of malaria.

## Background

Infection by intracellular pathogens is stressful for both the host as well as the pathogen. The pathogen which encounters a change in pH, temperature, degrading enzymes and ROS, up-regulates its heat shock protein (Hsp) repertoire post infection. Hsps are known antigens and many pathogen-encoded Hsp60s and Hsp70s are vaccine candidates [1].

*Plasmodium falciparum *causes cerebral malaria and 1-2 million deaths annually. Periodic fever is a hallmark of malaria exposing parasites to temperatures as high as 43°C in the patient. Survival and proliferation of the parasite under such heat stress conditions has triggered interest in examining parasite Hsps. Previous studies suggest that the parasite relies on its repertoire of Hsps, PfHsp90 in particular, to establish and grow during heat shock [2]. These insights however, have been gleaned from studies on laboratory cultures of the parasite. The information that exists about the roles of parasite chaperones in infected patients is limited to the antigenic nature of parasite chaperone Hsp70-I.

Hsp90 inhibition by geldanamycin in laboratory cultures has been demonstrated to be a successful method to inhibit parasite growth and a derivative of this drug is in phase III clinical trials as a tumor inhibitor [3-6]. However, the importance of Hsps in field isolates is not well explored. It is, therefore, important to combine studies from field isolates and laboratory strains to arrive at useful understanding of parasite growth and survival within the host.

A recent study has looked at the global transcriptome of *P. falciparum *isolated from infected patients and has reported novel observations about parasite *in vivo *biology [7]. Parasites that infect humans could be categorized into three distinct physiological states depending upon their gene expression profiles. Their analysis was based upon a comparison of gene expression profiles of these parasites with known pathways in *Saccharomyces cerevisiae*. Accordingly, parasites could be classified as belonging to clusters 1, 2 or 3. Cluster 1 representing starvation response; cluster 2 resembling 3D7 *in vitro *cultivated ring stages where glycolysis was the primary pathway and cluster 3 representing an environmental stress response. Important clinical and laboratory parameters of the patients in each cluster including age, parasitemia, hematocrit, did not vary [7]. Prior anti-malarial use and presence of gametocytes (rare) also did not differ between clusters. However patients from which cluster 3 parasites were obtained had significantly higher levels (P < 0.05, rank sum test) of inflammation including elevated IL6, IL10, C-reactive protein, TGF alpha levels and elevated temperature. This provides further support that cluster 3 parasites were derived from a higher environmentally stressed milieu compared to the other parasites. This study presented an opportunity to examine the relevance of parasite Hsps in clinical malaria. *P. falciparum *encodes for a large repertoire of molecular chaperones that constitute almost 2% of the parasite genome [8]. Chaperones of all major classes - Hsp100, Hsp90, Hsp70, Hsp60, Hsp40 and several small Hsps as well as their co-chaperones are present in the parasite.

This study reports the analysis of parasite-encoded chaperones, their interactors, pathways governed by them and implicate their role in clinical malaria. Interestingly, previously defined clusters highly correlate with expression levels of parasite-encoded chaperones. Further, chaperone interactomes among the patient samples show differential expression profiles. Strikingly Hsp90-dependent trafficking, anti-apoptotic and cell proliferation pathways seem to be up-regulated in a subset of patient samples accompanied by up-regulation of proteins involved in host remodeling processes. A group of patient samples which represent environmental stress response exhibited heterogeneity in chaperone transcript levels. Due to marked difference in the expression level of Hsp90 among these patients, an additional hierarchical clustering of these samples has been carried out on the basis of Hsp90 expression. Interestingly, this group of patient samples sub-clustered into two groups. By correlating the knowledge about chaperone function and their clients, with parasite transcriptome profiles in the patients, the contribution of chaperone driven pathways in defining the physiological states of the parasite in clinical malaria have been explored. Through their ability to influence parasite survival and virulence in the host, this study highlights molecular chaperones and Hsp90 in particular, as key mediators of parasite physiology in malaria patients.

## Methods

### Construction of cluster-wise chaperone networks

Transcriptome data based on microarray analysis of clinical isolates as well as 3D7 late ring stage was obtained from Daily *et al *[7]. A list of 103 chaperones was constructed by combining two pre-existing lists reported by Pavithra *et al *and Acharya *et al *[8,9]. Transcript level values were extracted for each of the 103 chaperones from the raw microarray data. A total of 43 patient samples were considered. There were no significant differences in the age, parasitemia, and the clinical presentation of the patients used in this study (See Additional file 1). Cluster 1 had eight patients, cluster 2 had 17 patients and cluster 3 had 18 patients. Transcript level for each gene in each patient was normalized against transcript level for the same gene in 3D7 late ring stage, to obtain fold up-regulation for that gene in each patient. An average of fold up-regulation of each gene was calculated for each cluster by summing the fold up-regulation for all the patients in all individual clusters, and subsequently dividing the total in each cluster by the number of patients in that cluster. For each gene, its average fold up-regulation was compared within the three clusters. A gene was said to be maximally expressed in the particular cluster in which its fold up-regulation was higher than its fold up-regulation in the other two clusters by at least "1 unit". The "1 unit" threshold was arrived at by taking into consideration fold up-regulation values of all genes and the kind of differences they exhibited within the three clusters. This information was used to construct clinical parasite-chaperone networks for each cluster separately using the software cytoscape [10]. Individual genes that were a part of the network were colour-coded according to their fold up-regulation or presence at basal levels comparable to 3D7 or less than 3D7 (Red: maximum up-regulation among the three clusters; orange: up-regulated but not maximum; yellow: at similar levels in the three clusters; pink: up-regulated as compared to 3D7 but least among the three clusters; green: present at basal levels comparable to 3D7 late ring stages or less than 3D7 late ring stages ).

### Analysis of PfHsp90-dependent pathways

The networks were then analysed to get an overall view of the effectors that were activated in the different physiological states of the parasite. The up-regulation and down-regulation of Hsp90-dependent pathways was determined by respective node expression patterns. A pathway was considered to be highly up-regulated, only if, the central hub i.e. Hsp90 and more than two primary nodes present in the pathway were expressed at the highest level as compared to 3D7 and other two clusters. A pathway was considered to be down-regulated if both the central hub and primary interactors were expressed at basal levels. If a pathway was controlled by more than one interacting nodes having different kinds of expression pattern, connected to a basal level central node, the pathway was considered to be up-regulated as compared to 3D7 but less than the other two clusters. If both the central node and the interactors of the pathway were up-regulated but not maximum, the pathway was considered to be up-regulated at an intermediate level. The colour codes are the same as that for networks.

### Sub-clustering of cluster 3

Cluster 3 was sub-clustered into clusters 3a and 3b on the basis of all genes using non-negative matrix factorization (NMF) using methods previously described [7]. Two clusters were chosen to allow each group size to be large and the cophenetic coefficient was robust at 0.9973. It was noted that Hsp90 was coherently undetected in cluster 3a, and present in cluster 3b samples suggesting an association of this gene and the dichotomous transcriptional patterns. Raw expression values for Hsp90 gene expression were used for the 18 samples in cluster 3 (See Additional file 2).

## Results

### Functionally related heat shock proteins are co-expressed in the same group of patients

**Figure 1:**
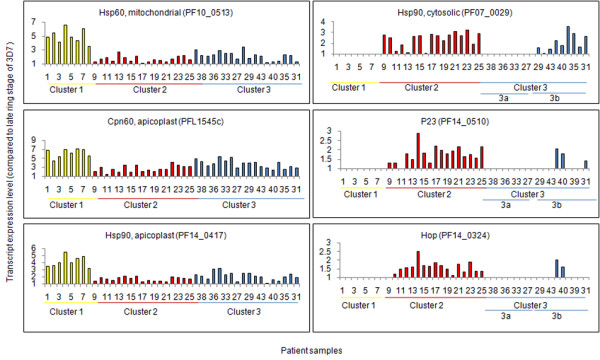
**Up-regulation of different chaperone classes in cluster 1 and cluster 2**. Organellar chaperones such as mitochondrial Hsp60, apicoplast Cpn60 and apicoplast Hsp90 are over-expressed in cluster 1. Cytosolic chaperones such as Hsp90_C, Hsp40 and Hsp90 co-chaperones are up-regulated in cluster 2. Cluster 3 shows heterogeneous expression of all genes. Yellow bars indicate cluster 1, red bars indicate cluster 2 and blue bars indicate cluster 3. Cytosolic Hsp90 was taken as a criterion for further clustering of cluster 3. Raw Hsp90 expression values for 18 patients of cluster 3 were used for NMF clustering into sub-clusters 3a and 3b. Clustering of PfHsp90 into two clusters suggest that PfHsp90 may be driving development of different states of the parasite.

Clinical isolates of *P. falciparum *have been reported to show distinct physiological states [7]. Re-analysis of transcriptome data for all parasite encoded chaperones from clinical isolates with 3D7 late ring stage as reference has been performed to analyse the chaperone expression patterns among the patients. Interestingly, organellar chaperones that are targeted to mitochondria or apicoplast, are found to be highly up-regulated in cluster 1 patients. Mitochondrial Hsp60 (Hsp60_M, PF10_0513), apicoplast Cpn60 (PFL1545c) and apicoplast Hsp90 (Hsp90_A, PF14_0417) transcripts are up-regulated in cluster 1 patients (Figure 1). Cytosolic chaperones such as cytosolic Hsp90 (Hsp90_C, PF07_0029), Hsp90 co-chaperones such as Hop (PF14_0324), p23 (PF14_0510), some cyclophilins and CHIP (PFE1370w), the Hsp70 homolog Cg4 (PF07_0033), some Hsp40 family members such as RESA- like proteins and subunits of TCP are highly up-regulated in cluster 2 patients and in some cluster 3 patients. Therefore cluster 2 shows high levels of expression of cytosolic chaperones whereas cluster 3 is heterogeneous in expression of these chaperones. These are either down-regulated or expressed at basal levels in cluster 1 patients. Cytosolic Hsp70 (Hsp70_C, PF08_0054) is found to be up-regulated only in some parasites of cluster 2. The most conspicuous difference in expression between clusters 1 and 2 is exhibited by Hsp90_C. Cluster 3 does not show uniform difference in fold expression of chaperones with respect to 3D7. Therefore, cluster 3 has been sub-clustered with respect to Hsp90_C. This results in two sub-clusters; 3a and 3b, which differ in expression levels of cytosolic chaperones, indicating that cytosolic chaperones have distinct expression profiles in distinct physiological states and sub-states of the parasite. Further, organellar chaperones do not follow this sub-clustering indicating that sub groups 3a and 3b may be specific for cytosolic chaperones (Figure 1). This also implies that clusters 2 and 3 represent related parasite physiology.

### Analysis of specific heat shock protein hubs in *P. falciparum*

Typically, cellular chaperones are present in complex with their co-chaperones and substrates. The parasite interactome has been utilized to identify chaperone-dependent pathways that are up-regulated or down-regulated in parasites from different patient clusters. Analysis of chaperone hubs yielded interesting results. Overall, cytosolic chaperone hubs are found to be most active in cluster 2 and cluster 3a and least active in cluster 1. Major active hubs are formed by Hsp90, the Hsp70 homolog Cg4 and many Hsp40 co-chaperones.

### PfHsp101

Hsp101 belongs to the Hsp100/Clp A/B chaperone family and is typically found as a component of protein translocating systems such as the type VI secretion systems in bacteria [11]. In the parasite, Hsp101 (PF11_0175) encodes for an ER-signal peptide containing protein. Recently, Hsp101 has been shown to be an integral component of the membrane translocon present in the parasitophorous vacuolar membrane involved in PEXEL-protein export [12]. The gene expression profile for PfHsp101 was analyzed in all the three patient clusters and is seen to be present at similar levels as in 3D7 in cluster 2 and is present at basal levels in clusters 1 and 3 (See Additional file 3). Further, the interactome for Hsp101 was constructed using experimentally known interactors and putative interactors (See Additional file 4) [9,12]. The interactome of Hsp101 consists of PTEX150 (PF14_0344), Exp2 (PF14_0678), Trx2 (MAL13P1.225), PTEX 88 (PF11_0067), 60S ribosomal subunit (PFE0845c) and a hypothetical protein (PFE0750c). Although the levels of Hsp101 in cluster 2 have remained similar to its levels in 3D7, 3 of the 4 known components of the PEXEL-translocon (PTEX150, Trx2 and PTEX88) are highly up-regulated in cluster 2. This is surprising since protein export is central to the life cycle of the parasite and is expected to be equally important in all parasites. However, the gene expression pattern of PfHsp101 suggests that protein export may be especially active in parasites that belong to cluster 2.

### PfHsp90 isoforms

**Figure 2:**
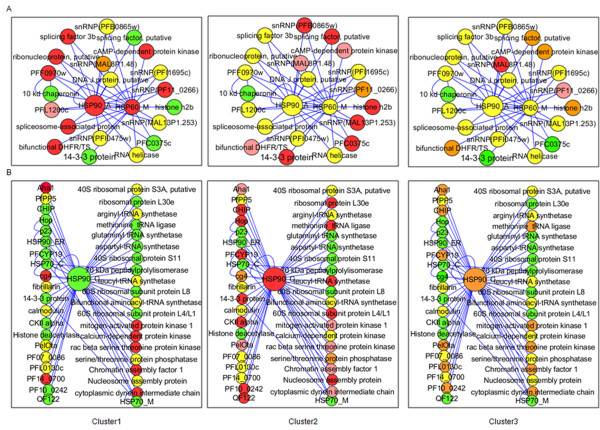
**Cluster-wise expression profile of Hsp90 and interacting partners**. (A) Apicoplast Hsp90 and its interacting partners are up-regulated in cluster 1 followed by cluster 2. Hsp90_A forms a hub with Hsp60_M (B) Cytosolic Hsp90 is up-regulated in cluster 2. Co-chaperones of Hsp90 such as p23, CHIP and Hop are also up-regulated in cluster 2. Colour codes are described below. (Red: maximum up-regulation among the three clusters; orange: up-regulated but not maximum; yellow: at similar levels in at least two clusters; pink: up-regulated as compared to 3D7 but least among the three clusters; green: present at basal levels comparable to 3D7 late ring stages or less than 3D7 late ring stages ).

The parasite contains only one cytosolic form of Hsp90 [3] and three more genes which code for organellar Hsp90 such as Hsp90_ER (Grp94, PF1070c, Hsp90 of endoplasmic reticulum), Hsp90_A and Hsp90_M (PF11_0188, Hsp90 of mitochondria). All these forms are expressed in patient-derived parasites (See Additional files 5 and 6). Among these, Hsp90_A shows up-regulation in cluster 1 and Hsp90_C shows up-regulation in cluster 2 and cluster 3a with respect to 3D7 late ring stages (Figure 1). Hsp90_A interacts with Hsp60_M and DNAJ domain, putative which are also maximum in cluster 1 (Figure 2A). Hsp90_A and Hsp60_M together interact with snRNPs, ribonucleotides, splicing factor and RNA helicases and seem to regulate the structure and function of spliceosome, the multi-mega ribonucleoprotein complex that performs the splicing of mRNA precursor in eukaryotes. The core of this complex formed by Hsp90_A and Hsp60_M, shows up-regulation in cluster 1 followed by clusters 2 and 3. The other components of this hub show a similar pattern across all three clusters. This indicates that Hsp90_A-Hsp60_M hub is involved in post-transcriptional regulation of protein function which is conserved in all physiologic states.

The cell uses two folding machineries: the Hsp70 machinery and the Hsp70-Hsp90 machinery [13]. Hsp90 exists as intermediate complex (Hsp90, Hsp70, Hop, Hsp40 and Hip) and mature complex (Hsp90, p23 and any immunophilin/PP5). Hsp90_C is up-regulated in cluster 2 and cluster 3a. Hop (Hsp70-Hsp90 organizing protein) is highest in cluster 2. Cyclophilins (PFC0975c) and p23, required for the conversion of early complex to mature complex, also show up-regulation in cluster 2 and cluster 3a (Figure1, See Additional file 6). Aha1 (PFC0270w), activator of Hsp90 ATPase, shows up-regulation in all parasites and is maximum in cluster 1 (See Additional file 6). Overall, most of the components of chaperone complex show up-regulation in cluster 2 and cluster 3a (See Additional file 5). Their increased expression in a group of patients may be an adaptive response to act as buffer to protect against the deleterious consequences of several stresses in the processes of progressive infection (Figure 2B).

A well-known group of Hsp90 co-chaperones is FK506 or cyclosporin binding family of immunophilins, involved in the formation of mature complex. There are 12 genes coding for immunophilins and cyclophilins in *P. falciparum*. Specific immunophilins have been shown to impart specificity to Hsp90 towards specific clients and finally towards a specific process. Differential expression of these immunophilins among different clusters could result in the activation of different pathways among them, which may be unique to the cluster and may be representative of disease status (See Additional file 5, Figure 2B). PfCYP19 is known to inhibit *Plasmodium *calcineurin (PfPP2B, PF14_0492) in the presence of cyclosporin [14]. PfFKBP35 (PFL2275c) possesses drug independent calcineurin-inhibitory activity [15] and is present at basal level in all clusters unlike calcineurin which is up-regulated several folds (Figure 2B). The higher expression of calcineurin may be a mechanism by which it evades inhibition by PfFKBP35.

Hsp90 influences multiple cellular processes. Hsp90 interacts with transcription factors and components of signal transduction and participates in diverse kinds of cellular functions. Almost 10% of the yeast proteome interacts with Hsp90 [16]. Apart from the published interactome, additional possible PfHsp90 interactors from the literature have been taken and analysed. Rab proteins are a large family in Ras superfamily of GTPases that are involved in membrane trafficking in eukaryotes. They alternate between GTP (active) form and GDP (inactive) forms [17,18]. Hsp90 is required for Rab recycling in the early exocytic pathways. Most of the interactors of Hsp90 involved in trafficking such as, Rab1 GTPases (PFE0690c), Rab5b (MAL13P1.51) and Rab6 (PF110461) show up-regulation in all the clusters. Only the key regulator GDI (PFL2060c) is highly expressed in cluster 2 along with Hsp90. This is suggestive of enhanced Hsp90-dependent trafficking in cluster 2.

**Figure 3:**
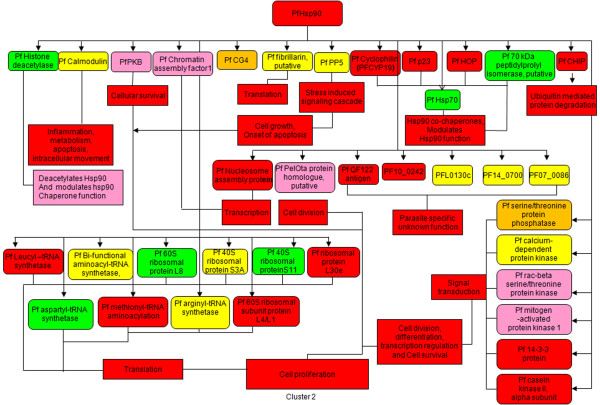
**Hsp90-dependent pathways are up-regulated in cluster 2**. Most Hsp90-dependent pro-cell survival pathways such as transcription, translation and cell signaling are up-regulated in cluster 2. (Red: maximum up-regulation among the three clusters; orange: up-regulated but not maximum; yellow: at similar levels in at least two clusters; pink: up-regulated as compared to 3D7 but least among the three clusters; green: present at basal levels comparable to 3D7 late ring stages or less than 3D7 late ring stages).

Hsp90 regulates the function of ribosome by maintaining the stability of 40S ribosomal subunit components and 60S ribosomal subunit components [19]. Hsp90 machinery is known to activate eIF2α kinases [20-22]. HRI is the major eIF2 α kinase responsible for the increased eIF2 α phosphorylation upon heat shock in erythroid cells [23]. PfPK4 (PFF1370w) is a protein kinase related to eIF-2alpha kinases. It is up-regulated in all clusters and peaks in cluster 2. Glycogen Synthase Kinase GSK3 (PFCO525c) is marginally up-regulated in clusters 1 and 3 and present at basal level in cluster 2 (Figure 3, See Additional file 7).

AKT (also known as Protein Kinase B; PKB) is a ser/thr kinase which inhibits pro-apoptotic signal. Hsp90 interacts with AKT and prevents its dephosphorylation by PP2A [24]. Homologs of Hsp90 (PF14_0029), PI3K (PFE0765w) and PP2A (PFI1245c) are present in *Plasmodium*. AKT is found to be up-regulated in all the clusters but shows maximum expression in cluster1, PI3K is up-regulated in all the clusters, high in clusters 2 and 3. Interestingly PP2A, the inhibitor of PKB kinase is highly up-regulated in cluster 1 followed by clusters 2 and 3. Hsp90 also regulates MAP kinase pathways, involved in cell proliferation, cell differentiation, cell movement and cell death. Raf homologs in *Plasmodium *are PFL0080c and PFB0520w. Homologues of MAP1/2 are PF14_0294 and PF11_0147 and MEK are Pfnek (PFL1370w). All the components of MAP kinase pathway are up-regulated in all the clusters but the pathway may be most active in cluster 2 because of the up-regulation of the master regulator Hsp90 in the same (Figure 3, Additional file 6). The up-regulation of AKT and MAP kinase pathway in cluster 2 implies an increased Hsp90-dependent anti-apoptotic pathway and cell proliferation pathway in this cluster (Figure 3).

### PfHsp70 family

**Figure 4:**
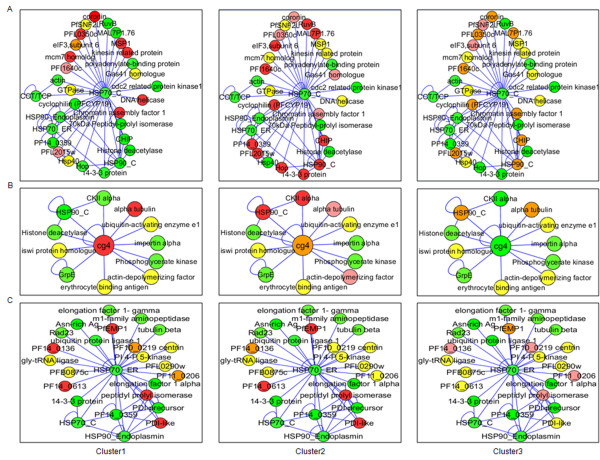
**Cluster-wise expression profile of Hsp70 and interacting partners**. (A) Hsp70-I (cytosolic) is expressed at basal levels in all three clusters while its interacting partners are differentially expressed. (B) Cg4 is up-regulated in cluster 1 while its interacting partners are differentially up-regulated. (C) Hsp70_ER is expressed at basal levels in all three clusters while different substrates are up-regulated in different clusters. (Red: maximum up-regulation among the three clusters; orange: up-regulated but not maximum; yellow: at similar levels in at least two clusters; pink: up-regulated as compared to 3D7 but least among the three clusters; green: present at basal levels comparable to 3D7 late ring stages or less than 3D7 late ring stages).

*P. falciparum *encodes for six genes that belong to the Hsp70 family-PF08_0054, PFI0875w, MAL7P1.228, PF11_0351, PF07_0033, and MAL13P1.540. Out of these six genes, transcript data for only four (PF08_0054, PFI0875w, PF11_0351 and PF07_0033) are found in the clinical isolates. Hsp70-I, the cytosolic isoform of parasite Hsp70 (Hsp70_C) has been extensively explored as an antigen and a vaccine candidate for malaria [25,26]. Hsp70_C transcript is found to be up-regulated only in some cluster 2 parasites. However, many of its interacting partners show up-regulation in different clusters. As with other chaperones Hsp70_C interactors that exhibited maximum transcript levels in cluster 2 patients are also found to be highly expressed in cluster 3 patients. On the other hand, cluster 1 patients show maximum expression for a different sub-group of interacting partners. It is possible that while the basal levels of Hsp70 isoforms present in the parasite are ample for parasite survival and virulence during infection, different Hsp70-dependent pathways are required by the parasite in different physiologic states (Figure 4A, See Additional file 8).

However, Cg4, a high molecular weight Hsp70, is highly expressed in all patients of cluster 2 (Figure 4B, See Additional file 8). Cg4 is homologous to the yeast Sse proteins. In yeast, the Sse proteins act as nucleotide exchange factors for Hsp70 and also perform independent roles as "holdases" that maintain substrates in folding competent states [27-29]. Sse are also implicated in PKA signaling and activity of the Hsp90 chaperone complex [30,31]. The function of Cg4 in *Plasmodium *life cycle has not yet been deciphered. Cg4 interacts with Hsp90_C and both are up-regulated in cluster 2, implying that holdase and PKA signaling functions of Cg4 are probably heightened in this cluster. In addition, although Hsp70_C is present at basal levels, higher levels of Cg4 may increase the flux through the Hsp70_C chaperone cycle by increasing its rate of nucleotide exchange. More importantly, these functions are much more enhanced in clinical malaria as compared to the lab strain.

Hsp70 ER isoform (Hsp70_ER, PFI0875w) and mitochondrial isoform (Hsp70_M, PF11_0351) are expressed at basal levels in all parasites. However, many interconnecting partners of these proteins are up-regulated differentially in the clusters. PF14_0359 is an Hsp40 protein that links Hsp70_C and Hsp70_ER and is up-regulated specifically in cluster 2 (Figure 4C). Since Hsp40s are known to confer substrate specificity to Hsp70, maturation of specific substrates by Hsp70 are promoted by regulation of Hsp40 levels in different physiologic states. The substrates for Hsp70_C as well as Hsp70_ER are also different in the different clusters.

### PfHsp40 co-chaperones

The Hsp40 family constitutes the largest subset of chaperones in *P. falciparum *with 44 genes encoding the J domain [8]. Hsp40 are the only chaperones that contain the *Plasmodium *export element (PEXEL) [32] and have been postulated to have regulatory roles in the parasite and host-remodeling activities in the infected erythrocyte. Hsp40s are known to modulate the Hsp70 ATPase activity and confer substrate specificity to their Hsp70 partner [33]. Out of the 44 Hsp40s in the parasite, 28 are up-regulated compared to laboratory cultures in the three physiologic states, several contain PEXEL motifs (See Additional files 9 and 10). Out of the 28 six are RESA or RESA-like proteins. Cluster 1 over-expresses a different sub-population of RESA as compared to clusters 2 and 3 (See Additional file 9). Clusters 2 and 3 over-express more number of RESA/RESA-like proteins as compared to cluster 1. Many unique hypothetical proteins are also up-regulated in the three clusters (See Additional file 9). The precise roles of Hsp40 in the parasite are not known. Apart from RESA, the only report on parasite Hsp40 function is on PfJ4 that has been shown to interact with parasite Hsp70_C and is up-regulated during heat stress [34]. Interestingly, PfJ4 (PFL0565w) is found to be up-regulated in cluster 2, this protein interacts with PfGCN20, which is at basal levels across diverse disease states. Although substrates of PfJ4 have not been reported, analysis of transcriptome suggests PfGCN20 and PF11_0225 could be PfJ4 substrates requiring chaperoning during heightened stress in the parasite, as represented by cluster 2.

**Figure 5:**
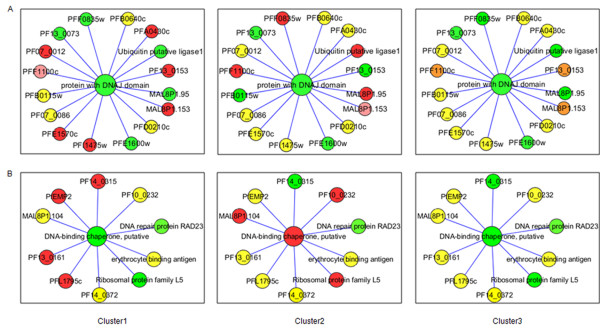
**Cluster-wise expression profile of Hsp40 and interacting partners**. (A) Protein with DnaJ domain (PFE1605w) is expressed at basal levels in all three clusters but different interacting partners are up-regulated in clusters 1 and 2. Cluster 3 shows intermediate expression of the interacting partners. (B) DNA binding chaperone (PFL0815w) is up-regulated in cluster 2 and some of its interactors are up-regulated in clusters 1 or 2. (Red: maximum up-regulation among the three clusters; orange: up-regulated but not maximum; yellow: at similar levels in at least two clusters; pink: up-regulated as compared to 3D7 but least among the three clusters; green: present at basal levels comparable to 3D7 late ring stages or less than 3D7 late ring stages).

Many PfHsp40s form very small hubs within the parasite interactome, interacting with one or two partners at a time. As stated above, different RESA are over-expressed in clusters 1 and 2. RESA have been predicted to interact with each other, forming hetero-oligomers. Therefore, different RESA hetero-oligomers are present in different disease states. Sec63 is a transmembrane endoplasmic reticulum (ER) Hsp40 that is involved in translocation of signal peptide containing proteins into the ER. The Sec63 homolog in *P. falciparum *(PF13_0102) is up-regulated in cluster 2. Its interactors MAL8P1.153 and PFI1780w, both hypothetical proteins, are over-expressed in clusters 1 and 2 respectively. Only two Hsp40s form sizeable hubs in the parasite interactome: PFE1605w and PFL0815w. PFE1605w is expressed at basal levels across all the three clusters however different interactors of this Hsp40 are over-expressed in different clusters (Figure 5A). All interactors of PFE1605w are hypothetical proteins, except PFI1475w (merozoite surface protein 1, precursor). It shares MAL8P1.153 as a common interacting partner with Sec63 homolog. Interestingly, MAL8P1.153 is up-regulated in cluster 1 where Sec63 is not over expressed. Sec63 is up-regulated in cluster 2 where MAL8P1.153 is not over-expressed. Therefore PFE1605w probably has a dual role to play in the two different clusters where two different substrates are up-regulated. PFL0815w has been annotated as a DNA binding chaperone because it contains two DNA binding domains in its C-terminus. It is homologous to the mammalian DnaJ homolog MPP11 [35] which is involved in translation in mammalian cells. Although almost all interactors of PFL0815w are over-expressed in all the patients, the central node of this hub is up-regulated only in cluster 2 (Figure 5B), again hinting at a role in cluster 2 specific stress response pathway, similar to Hsp90, Cg4 and PfJ4. In particular, PFL0815w interacts with PF14_0230 (Ribosomal protein family L5, putative) which is also over-expressed in cluster 2 indicating that its translation related functions may be up-regulated in cluster 2.

Except for RESA, functions of other exported Hsp40s are not known. A recent report has indicated that two exported Hsp40s in the parasite (PFE0055c and PFA0660w) are present in cholesterol-rich vesicles in the erythrocyte that the authors have christened "J-dots" and may be involved in protein trafficking in the erythrocyte cytosol [36]. Analysis of gene expression of these two Hsp40 genes across the clusters reveals that although both the proteins are up-regulated in comparison to the laboratory strain 3D7, PFE0055c is highest in cluster 2 whereas PFA0660w is relatively up-regulated in cluster 1 (See Additional file 11). These two proteins share a high degree of sequence homology [8,37] and may perform related functions in two different groups of parasites.

## Discussion

Heat shock episodes comprise an obligatory part of the parasite life cycle occuring first when the parasite undergoes an over 10^0^C shift from its poikilothermic mosquito vector to the human host and second, during fever in the host. In fact, the parasite dedicates almost 2% of its genome to encoding chaperones, 103 in total. All the major heat shock protein classes, such as Hsp100, Hsp90, Hsp70, Hsp60 and Hsp40, have been described and have been found to have roles in the asexual stages of the parasite. Hsp90 in particular has been shown to be critical for parasite growth in culture and abrogation of Hsp90 function by geldanamycin results in inhibition of parasite growth [3]. Almost all the knowledge about molecular chaperones in the parasite life cycle is derived from experiments carried out in the laboratory cultures. Although PfHsp70-I has been implicated as an important antigen and vaccine candidate [25,26], importance of the heat shock machinery in the parasite life cycle during human infection has not been addressed. A recent study by Daily *et al. *[7] shed important light on the physiology of clinical isolates of *P. falciparum *during malaria infection. One of the physiologic states of the parasite exhibited a distinct environmental stress response-like gene expression profile. In order to analyse it more closely, transcript levels of 103 chaperones from clinical isolates has been examined. It is found that most of the chaperones are highly over-expressed in clinical parasite isolates and display a specific pattern of expression. This indicates that parasite molecular chaperones are important in malaria-infected individuals. Cluster 1 demonstrates over-expression of most mitochondrial and apicoplast chaperones whereas cluster 2 over-expresses many cytosolic chaperones. Interestingly, cluster 3, which does not have a unique chaperone profile, consists of two sub-clusters, one of which over-expresses cytosolic chaperones.

Chaperones almost always act in concert with their co-chaperones and there is cross talk between different chaperone pathways. Hsp100, Hsp90, Hsp70 and Hsp40 families, that are up-regulated in distinct parasite groups during infection of the human host, have been analysed. Out of these chaperone families, Hsp90 and some members of the Hsp40 family show over-expression in some patients.

The role of Hsp101 as a core component of the PEXEL-translocon in the parasitophorous vacuolar membrane has only recently been uncovered [12]. Although the levels of Hsp101 itself is identical to its levels in 3D7, the other components of the PEXEL translocon are highly up-regulated in cluster 2. This implies high protein-export activity in cluster 2 parasites as compared to other parasites.

Interestingly, Hsp90 (Hsp90_C) shows maximum up-regulation in cluster 2 and in a specific sub-population in cluster 3 (3b). Hsp90 co-chaperones such as Hop, p23 and CHIP are also up-regulated in cluster 2 and cluster 3b, similar to Hsp90 itself. As a result, many Hsp90-dependent pathways such as trafficking and signaling, appear to be most active in cluster 2 followed by cluster 3b. Inhibitors of probable clients of Hsp90 have also been shown to inhibit parasite growth in culture [3]. It is possible to exploit cluster specific co-chaperones in designing therapeutic strategies to overcome malaria. Furthermore, the Hsp90-dependent anti-apoptotic and pro-survival pathways that are up-regulated in cluster 2 favour parasite survival.

Out of the five Hsp70 genes, only Cg4 (Hsp110 family) is over-expressed in cluster 2. The other three Hsp70 isoforms are expressed at basal levels in all the three clusters. PfHsp70-I (Hsp70_C), a well-known antigen in malaria infections, is over-expressed only in some cluster 2 parasites. Possibly, the basal transcript levels of Hsp70 genes are abundant enough for parasite survival.

A recurring theme in parasite up-regulation of chaperone dependent pathways is that, although the core chaperone of a hub itself is not highly up-regulated in any specific cluster, its interacting partners such as co-chaperones and substrates are differentially up-regulated in different clusters. For instance Hop (Hsp70-Hp90 organizing protein) that brings Hsp70 and Hsp90 together by interacting with each through different TPR domains, is highly up-regulated in cluster 2 indicating that the Hsp70-Hsp90 machinery is most prominent in this group of patients. Similarly, many Hsp40 chaperones are up-regulated in either of the clusters providing a clue as to how this class of chaperones shapes parasite physiological states. Distinct subsets of Hsp40 are up-regulated in clusters 1 and 2. Cluster 3b shows up-regulation of RESA which is also up-regulated in cluster 2.

Hsp40s regulate the activity of the Hsp70 N-terminal ATPase domain and confer substrate specificity to them. Although Hsp70 genes are themselves not highly up-regulated, different clusters have different substrates which are activated due to modulation of specific Hsp40s. Many parasite Hsp40s also contain the PEXEL motif for transport to the erythrocytic compartment. Some of these are RESA and RESA-like Hsp40 which are known to stabilize the infected erythrocyte cytoskeleton during heat stress [38]. Recently, two exported Hsp40s have been shown to form vesicles known as "J-dots" in the erythrocyte cytosol [36]. One of these, PFE0055c, is significantly up-regulated in cluster 2 and the other, PFA0660w, is slightly up-regulated in cluster 1. The association of specific Hsp40 proteins with each cluster indicates that cluster 1 and 2 parasites utilize different pathways that are unique to *P. falciparum *and distinct from the host, implicating Hsps40 as novel drug targets in the parasite. In the reverse, their up-regulation in specific clusters, hints at the functions of the parasite Hsp40s.

Up-regulation in cluster 1 may indicate a role in starvation response of the parasite and up-regulation in cluster 2 may indicate a role in the cytosolic stress response pathway. Whether these expression profiles drive or "chaperone" the physiologic states remains to be answered.

It is generally assumed that parasites up-regulate chaperones in response to the hostile environment encountered upon infection of the host. This study reveals that it is not a generalized response and sub-populations of the same parasite, in this case *P. falciparum*, up-regulate different groups of chaperones in the host. This reflects fine-tuning of parasite stress responses, at times organellar and at times cytosolic, that depends on hitherto unknown host influences.

## Conclusion

In summary, analysis of chaperone networks in parasite samples from patients has been carried out by utilizing transcriptome data from patient samples in order to construct cluster-specific chaperone networks in clinical malaria parasite. Cluster1 parasites have been shown to be distinct from cluster 2 and cluster 3 parasites [7]. Based on their chaperone expression patterns parasites can be categorized into three groups. This suggests that the development of different parasite groups can be influenced by their chaperone profiles. Further, cluster 3, which exhibited an environmental stress response, can be further sub-clustered on the basis of Hsp90 gene expression. This is important since the sub-clusters also show a difference in up-regulation of cytosolic and organellar chaperones. Cluster 1 shows up-regulation of mitochondrial and apicoplast chaperones where as cluster 2 and cluster 3b show up-regulation of cytosolic chaperones. Further, PfHsp90 (Hsp90_C) dependent pathways that are up-regulated in cluster 2 skew the cell towards survival and proliferation. The existence of parasites in the human host in different physiological states and sub-states immediately increases the complexity of host-parasite interactions. In addition, the issue of drug efficacy in malaria infections has also become more complicated since parasites with different gene expression profiles are treated with the same drugs. This is an important step towards understanding host-parasite interactions and subsequently, treatment of severe malaria.

## Conflict of interests

The authors declare that they have no competing interests.

## Authors' contributions

R.P and P.A: Conceptualized and designed the study; acquired and analysed data; wrote the manuscript. S.C: acquired and analysed data. JPD: acquired and analysed data, critically read the manuscript. UT: conceptualized and designed the study; acquired data, drafted the manuscript and coordinated the study. All authors read and approved the final manuscript.

## Supplementary Material

Additional file 1 **Cluster-wise information of patients**. There were no significant differences in the age, parasitemia, and clinical presentation (all patient had fever and symptoms for malaria) of the patients used in this study. Ring stages predominated in the peripheral blood and no gametocytes were observed. Samples were collected prior to drug treatment. The only statistically significant values (*P *< 0.05) were found in cluster 3 is associated with significantly elevated inflammation markers, including duration of illness and body temperature. "*" represent statistically significant values. This information is adapted from Daily *et al *[7]. Click here for file

Additional file 2 **Cluster 3 can be divided into two sub-clusters**. Cytosolic Hsp90 was taken as the criterion for sub-clustering of cluster 3. Raw expression values for 18 patients of cluster 3 were used for NMF clustering. Clustering of cytosolic Hsp90 into two clusters suggests that cytosolic Hsp90 may be driving development of different states of parasite. Click here for file

Additional file 3 **Cluster-wise expression profile of Hsp101**. Only few patients in cluster 2 show up-regulation of Hsp101 as compared to 3D7 ring stage. However, average value for gene expression of Hsp101 in cluster 2 is identical to that of 3D7. Yellow bars indicate cluster 1, red bar indicates cluster 2 and blue bar indicates cluster 3. Click here for file

Additional file 4 **Cluster-wise expression pattern for Hsp101 interactors Hsp101 is present at basal levels in cluster 2**. However, the interactors of Hsp101, shown to be components of the PEXEL translocon are up-regulated in cluster 2. (Red: maximum up-regulation among the three clusters; orange: up-regulated but not maximum; yellow: at similar levels in at least two clusters; pink: up-regulated as compared to 3D7 but least among the three clusters; green: present at basal levels comparable to 3D7 late ring stages or less than 3D7 late ring stages). Click here for file

Additional file 5 **List of cluster wise distribution of Hsp90 class of chaperones and its co-chaperones**. Showing the cluster wise distribution of Hsp90 class of chaperones and its co-chaperones. (+) represents the presence and (-) represents the absence of chaperone in a particular cluster. Click here for file

Additional file 6 **Representative graphs of transcript level profile of Hsp90 and its co-chaperones**. Hsp90 of mitochondria and endoplasmic reticulum shows basal level of expression throughout the entire patient. Most of the co-chaperones like p23, Hop, PP5 and peptidyl-prolyl isomerase show clustering similar to cytosolic Hsp90. This supports the association of specific chaperone pattern with each state. Yellow bars indicate cluster 1, red bars indicate cluster 2 and blue bars indicate cluster 3. Click here for file

Additional file 7 **Cluster-wise expression profile of proteins involved in Hsp90-dependent pathways**. Most of the proteins show up-regulation in all samples, suggesting that decision of a pathway to be up-regulated or down-regulated may depend on expression profile of Hsp90 and its co-chaperones, which are up-regulated in cluster 2. Yellow bars indicate cluster 1, red bars indicate cluster 2 and blue bars indicate cluster 3. Click here for file

Additional file 8 **Cluster-wise expression profile of Hsp70 family proteins**. Hsp70 shows basal level of expression in all physiological states hinting there is no specific pattern associated with it. Cg4, one of the members of Hsp70 family, shows up-regulation in cluster 2. Yellow bars indicate cluster 1, red bars indicate cluster 2 and blue bars indicate cluster 3. Click here for file

Additional file 9 **List of cluster-wise distribution of Hsp40 class of chaperones**. Showing cluster wise distribution of Hsp40 class of chaperones. (+) represents the presence and (-) represents the absence of chaperone in particular cluster. The proteins indicated in bold are PEXEL-containing Hsp40s. Click here for file

Additional file 10 **Cluster-wise expression profile of Hsp40**. Most of the members of Hsp40 like RESA, RESA like protein, Pfj4 among others show up-regulation in cluster 2 and sub-clustered into cluster 3a and 3b. Yellow bars indicate cluster 1, red bars indicate cluster 2 and blue bars indicate cluster 3. Click here for file

Additional file 11 **Cluster-wise expression profile for Hsp40s which form J-dots**. One of the Hsp40s which forms J-dots, PFE0055c, is significantly up-regulated in cluster 2 while the other, PFA0660w, is slightly up-regulated in cluster 1. Yellow bars indicate cluster 1, red bars indicate cluster 2 and blue bars indicate cluster 3. Click here for file
