# Supplementary material for: Chaperone expression profiles correlate with distinct physiological states of Plasmodium falciparum in malaria patients
Source: Malar J. 2010 Aug 19;9:236. doi: 10.1186/1475-2875-9-236 (PMC2933700; doi:10.1186/1475-2875-9-236)
Supplement: Additional file 1 — Cluster-wise information of patients. There were no significant differences in the age, parasitemia, and clinical presentation (all patient had fever and symptoms for malaria) of the patients used in this study. Ring stages predominated in the peripheral blood and no gametocytes were observed. Samples were collected prior to drug treatment. The only statistically significant values (P < 0.05) were found in cluster 3 is associated with significantly elevated inflammation markers, including duration of illness and body temperature. "*" represent statistically significant values. This information is adapted from Daily et al [7]. [file 1475-2875-9-236-S1.DOC]

**Additional file 1. Cluster-wise information of patients**

| **Variable** | **Cluster 1 (n=8)** | **Cluster 2 (n=17)** | **Cluster 3 (n=18)** |
| --- | --- | --- | --- |
| Age | 5 (2-15) | 6 (3-6) | 7 (4-15) |
| Parasitaemia (%) | 2 (1-8) | 4 (2-6) | 3 (2-9) |
| Glucose (mg/dl) | 108 (94-113) | 99 (88-121) | 101(54-113) |
| Haematocrit | 39 (30-45) | 33(29-36) | 30 (27-35) |
| Days ill | 3 (3-3) | 3 (2-3.8) | 4* (3-6) |
| Temperature (ᵒC) | 38 (37-40) | 38 (37-39) | 39* (38-40) |
| Drug pressure | - | - | - |
| Peripheral blood smear examination  Rings  Gametocytes | +  - | +  - | +  - |
