# Supplementary figures and images for: Chaperone expression profiles correlate with distinct physiological states of Plasmodium falciparum in malaria patients
Source: Malar J. 2010 Aug 19;9:236. doi: 10.1186/1475-2875-9-236 (PMC2933700; doi:10.1186/1475-2875-9-236)

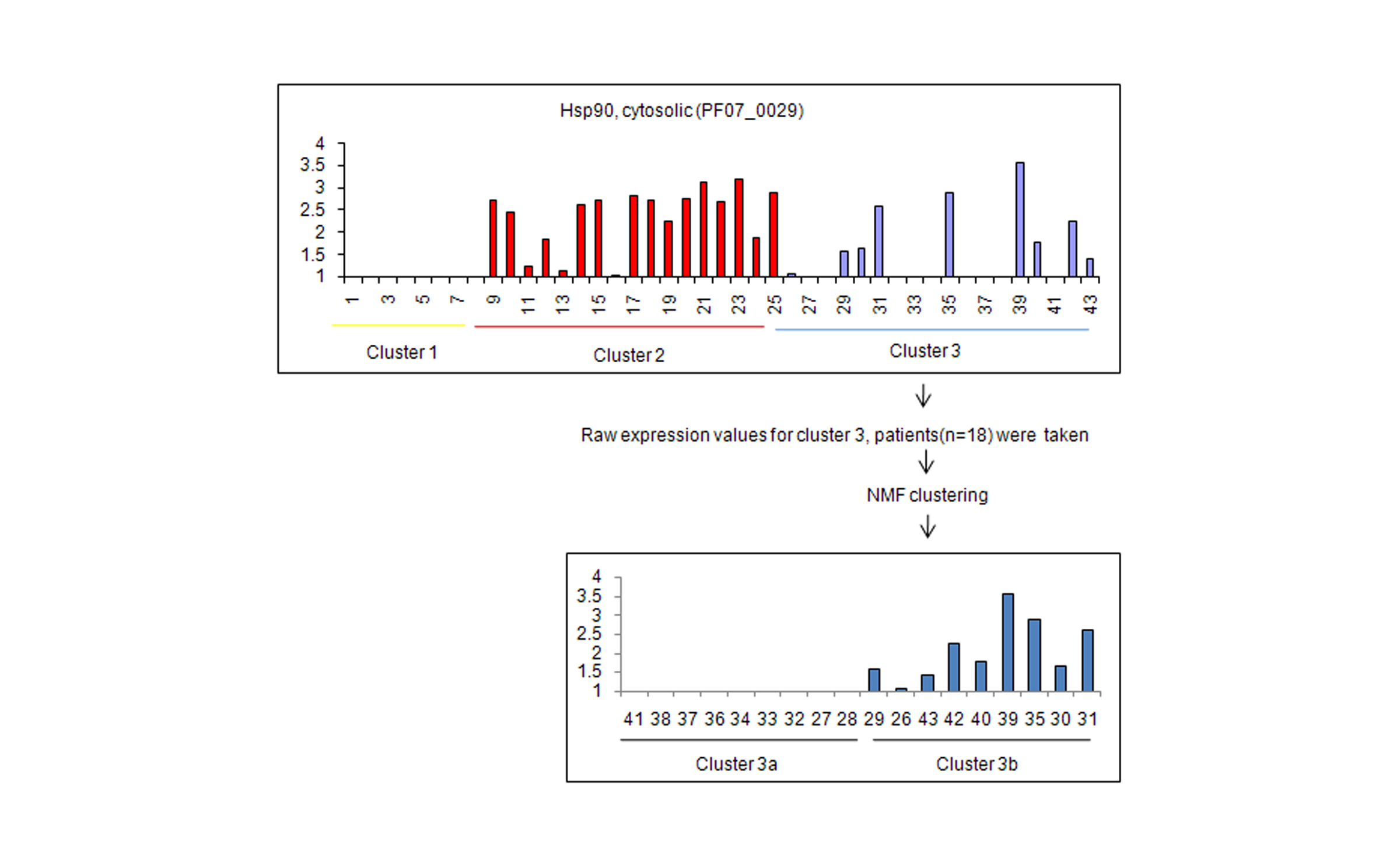

Supplement: Additional file 2 — Cluster 3 can be divided into two sub-clusters. Cytosolic Hsp90 was taken as the criterion for sub-clustering of cluster 3. Raw expression values for 18 patients of cluster 3 were used for NMF clustering. Clustering of cytosolic Hsp90 into two clusters suggests that cytosolic Hsp90 may be driving development of different states of parasite. [file 1475-2875-9-236-S2.TIFF]

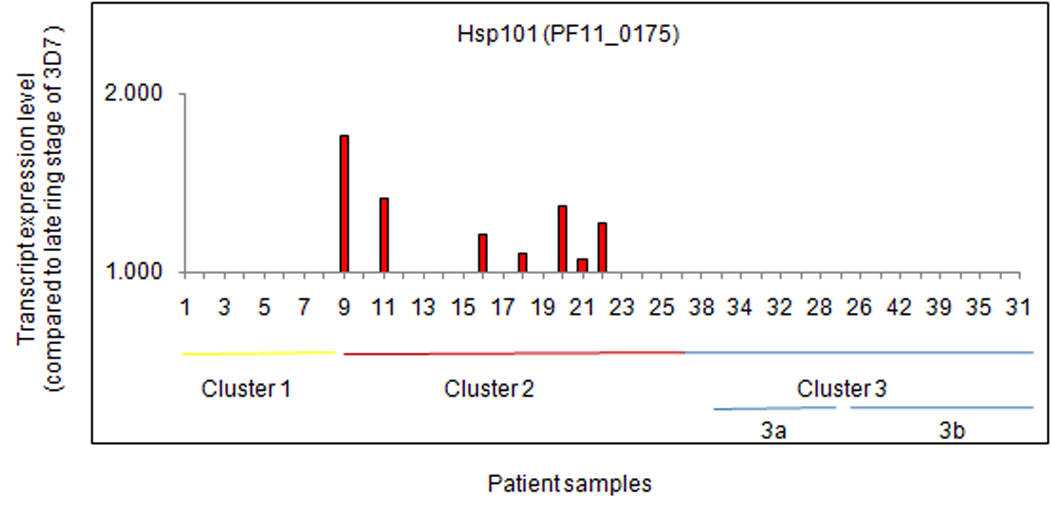

Supplement: Additional file 3 — Cluster-wise expression profile of Hsp101. Only few patients in cluster 2 show up-regulation of Hsp101 as compared to 3D7 ring stage. However, average value for gene expression of Hsp101 in cluster 2 is identical to that of 3D7. Yellow bars indicate cluster 1, red bar indicates cluster 2 and blue bar indicates cluster 3. [file 1475-2875-9-236-S3.TIFF]

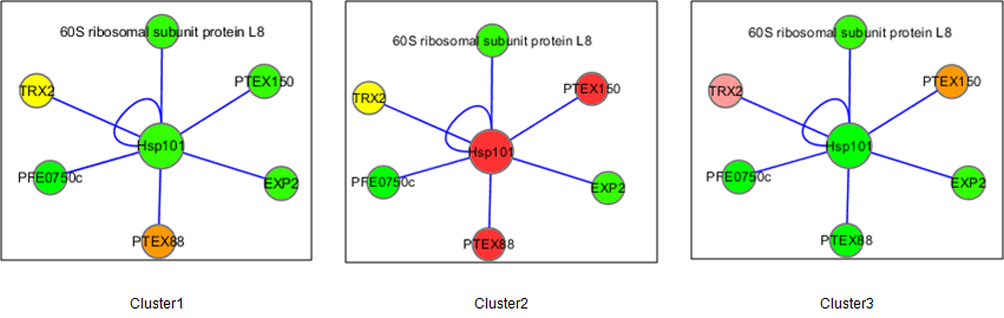

Supplement: Additional file 4 — Cluster-wise expression pattern for Hsp101 interactors Hsp101 is present at basal levels in cluster 2. However, the interactors of Hsp101, shown to be components of the PEXEL translocon are up-regulated in cluster 2. (Red: maximum up-regulation among the three clusters; orange: up-regulated but not maximum; yellow: at similar levels in at least two clusters; pink: up-regulated as compared to 3D7 but least among the three clusters; green: present at basal levels comparable to 3D7 late ring stages or less than 3D7 late ring stages). [file 1475-2875-9-236-S4.TIFF]

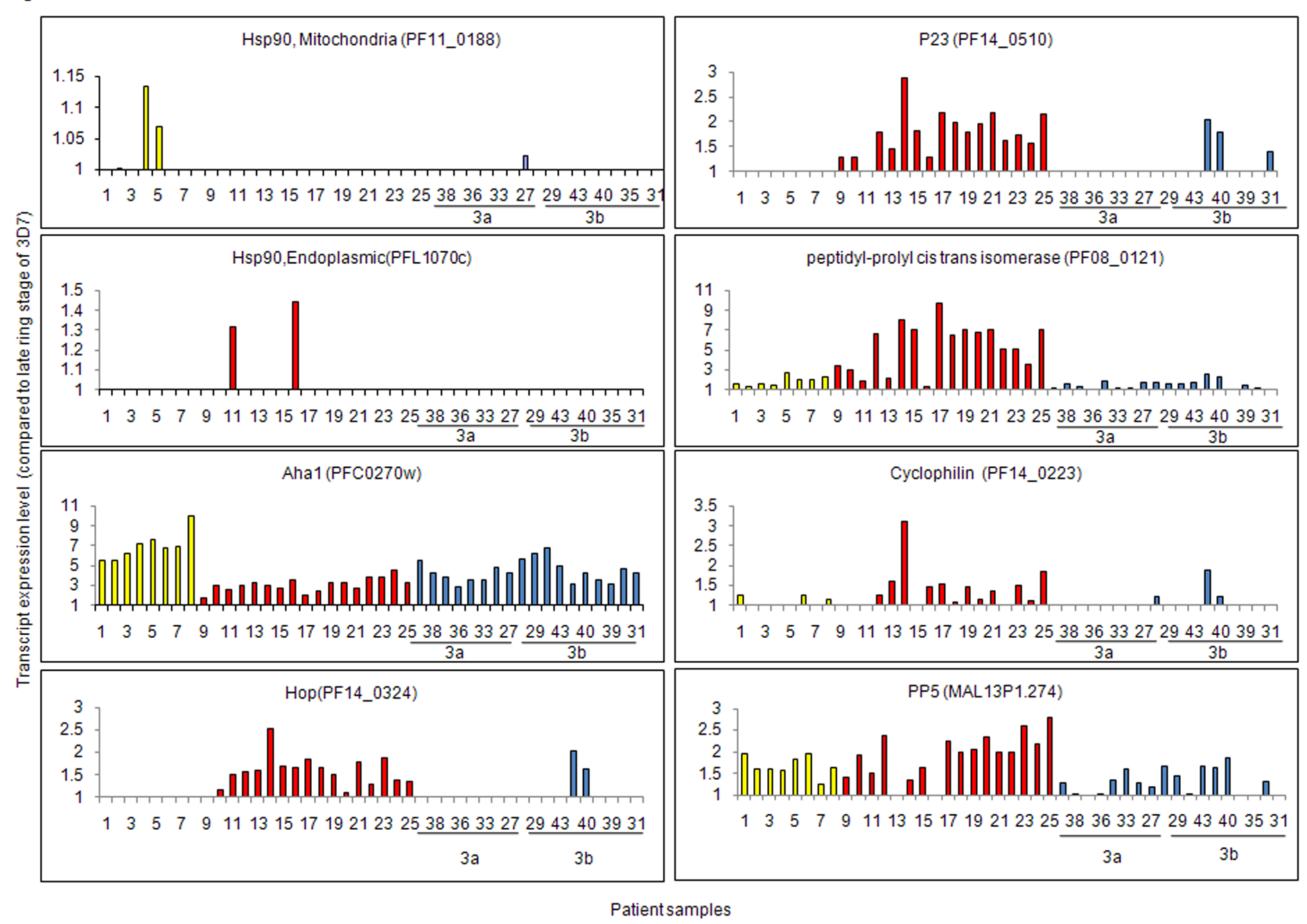

Supplement: Additional file 6 — Representative graphs of transcript level profile of Hsp90 and its co-chaperones. Hsp90 of mitochondria and endoplasmic reticulum shows basal level of expression throughout the entire patient. Most of the co-chaperones like p23, Hop, PP5 and peptidyl-prolyl isomerase show clustering similar to cytosolic Hsp90. This supports the association of specific chaperone pattern with each state. Yellow bars indicate cluster 1, red bars indicate cluster 2 and blue bars indicate cluster 3. [file 1475-2875-9-236-S6.TIFF]

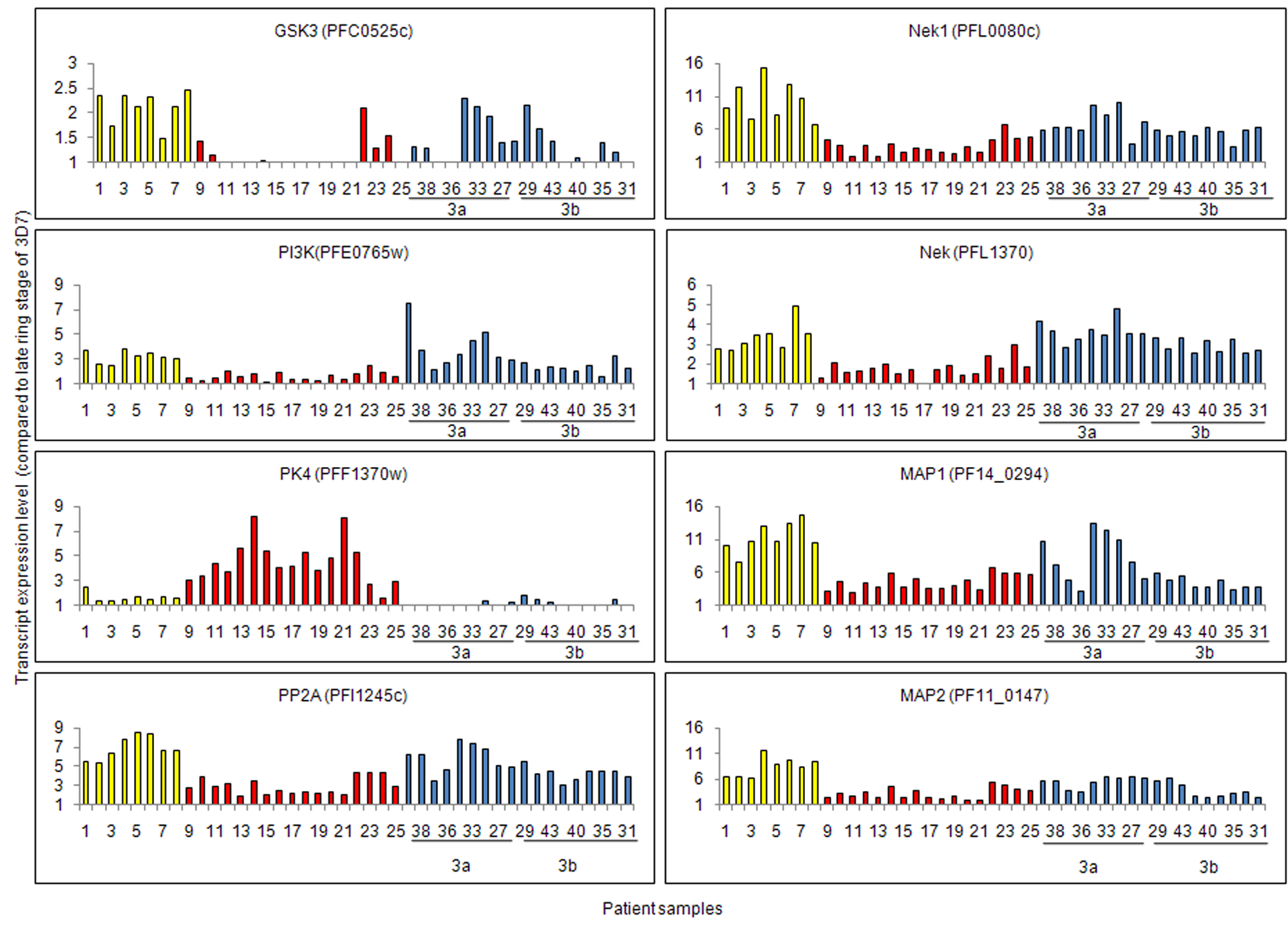

Supplement: Additional file 7 — Cluster-wise expression profile of proteins involved in Hsp90-dependent pathways. Most of the proteins show up-regulation in all samples, suggesting that decision of a pathway to be up-regulated or down-regulated may depend on expression profile of Hsp90 and its co-chaperones, which are up-regulated in cluster 2. Yellow bars indicate cluster 1, red bars indicate cluster 2 and blue bars indicate cluster 3. [file 1475-2875-9-236-S7.TIFF]

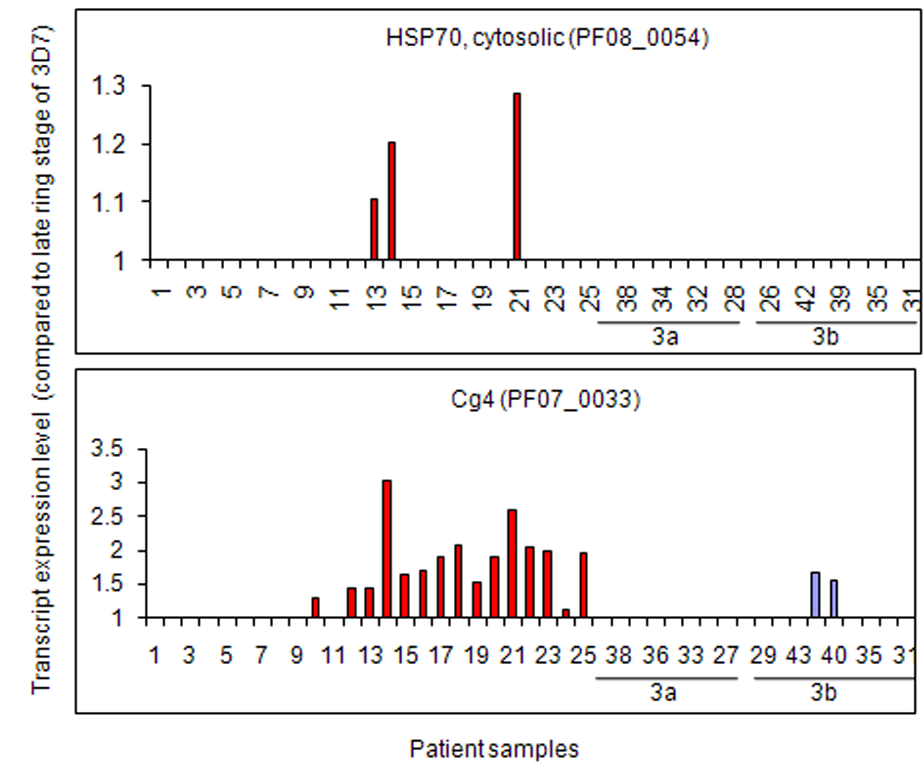

Supplement: Additional file 8 — Cluster-wise expression profile of Hsp70 family proteins. Hsp70 shows basal level of expression in all physiological states hinting there is no specific pattern associated with it. Cg4, one of the members of Hsp70 family, shows up-regulation in cluster 2. Yellow bars indicate cluster 1, red bars indicate cluster 2 and blue bars indicate cluster 3. [file 1475-2875-9-236-S8.TIFF]

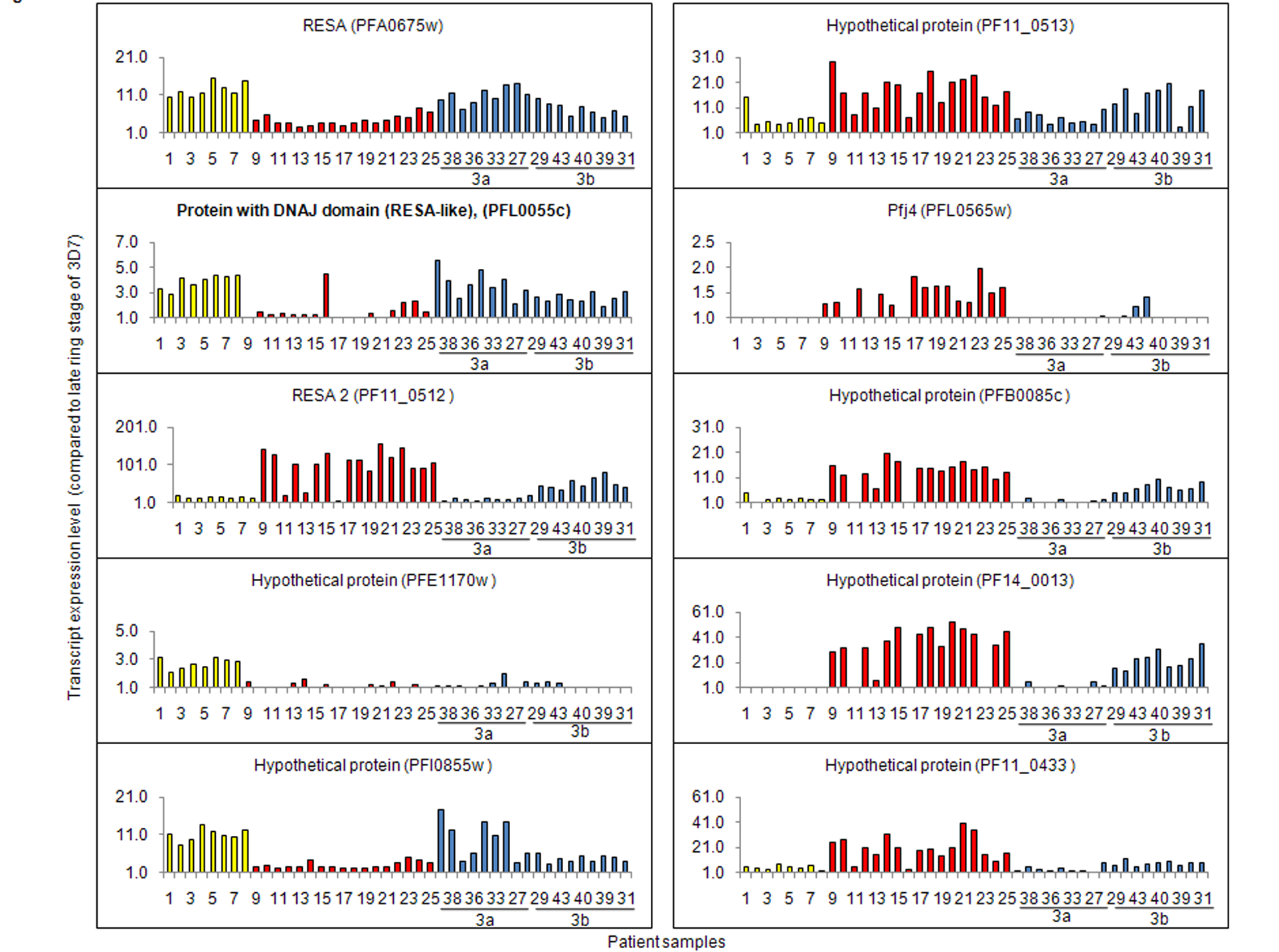

Supplement: Additional file 10 — Cluster-wise expression profile of Hsp40. Most of the members of Hsp40 like RESA, RESA like protein, Pfj4 among others show up-regulation in cluster 2 and sub-clustered into cluster 3a and 3b. Yellow bars indicate cluster 1, red bars indicate cluster 2 and blue bars indicate cluster 3. [file 1475-2875-9-236-S10.TIFF]

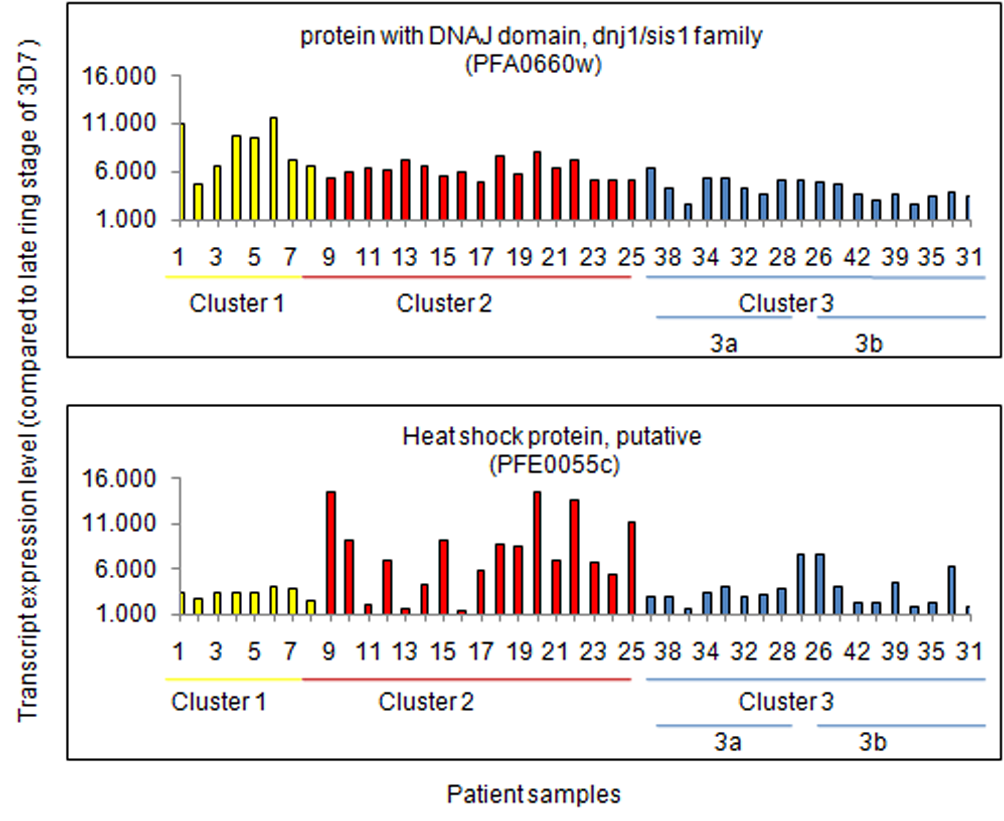

Supplement: Additional file 11 — Cluster-wise expression profile for Hsp40s which form J-dots. One of the Hsp40s which forms J-dots, PFE0055c, is significantly up-regulated in cluster 2 while the other, PFA0660w, is slightly up-regulated in cluster 1. Yellow bars indicate cluster 1, red bars indicate cluster 2 and blue bars indicate cluster 3. [file 1475-2875-9-236-S11.TIFF]
