# Supplementary material for: Chaperone expression profiles correlate with distinct physiological states of Plasmodium falciparum in malaria patients
Source: Malar J. 2010 Aug 19;9:236. doi: 10.1186/1475-2875-9-236 (PMC2933700; doi:10.1186/1475-2875-9-236)
Supplement: Additional file 5 — List of cluster wise distribution of Hsp90 class of chaperones and its co-chaperones. Showing the cluster wise distribution of Hsp90 class of chaperones and its co-chaperones. (+) represents the presence and (-) represents the absence of chaperone in a particular cluster. [file 1475-2875-9-236-S5.DOC]

**Additional file 5. List of cluster-**wise distribution of Hsp90 class of chaperones and its co-chaperones

| **Protein** | **1** | **2** | **3a** | **3b** | **Processes** |
| --- | --- | --- | --- | --- | --- |
| **Hsp90**  Hsp90_C (PF07_0029)  Hsp90_A (PF14_0417)  Hsp90_M (PF11_0188)  Hsp90_ER (PF1070c)  **Hsp90 co-chaperones**  Hop    Aha1(PFC0270w)  p23(PF14_0510)      PP5 (PF13_0294)  CHIP (PFE1370w)  PfCYP19 (PFCO975c)  Cyclophilin (PF14_0223)  FKBP (PF11_0124)  Cyclophilin (PF13_0190)  Cyclophilin (PF13_0122)  Cyclophilin (PFL0735w)  FKBP35 (PFL2275c)  Cyclophilin (PFE1430c)  Cyclophilin (PFL0120c)  Large cyclophilin like  protein (PFI1490c)  Peptidyl-prolyl cis-trans  isomerase  Peptidyl-prolyl cis-trans  isomerase | -  **+**  -  -  -  **+**  **-**  **+**  **-**  **-**  **-**  **+**  **+**  **+**  **+**  **-**  **+**  **+**  **+**  **-**  **+** | **+**  -  -  -  **+**  **+**  **+**  **+**  **+**  **+**  **+**  **+**  **+**  **+**  **+**  **-**  **+**  **+**  **+**  **+**  **+** | -  -  -  -  -  **+**  **-**  **+**  **-**  **-**  **-**  **+**  **+**  **+**  **+**  **-**  **+**  **+**  **+**  **-**  **+** | **+**  -  -  -  **+**  **+**  **+**  **+**  **+**  **+**  **+**  **+**  **+**  **+**  **+**  **-**  **+**  **+**  **+**  **+**  **+** | Translation, Cell proliferation, Signal transduction etc.  Help in substrate maturation, Adaptor for Hsp90/Hsp70  Regulator of Hsp90 ATPase activity  ATPase activator, Participate in substrate maturation, Binding to the N-terminal closed conformation, Inhibit Hsp90 ATPase activity  Dephosphorylation of Hsp90  Tagging proteins for degradation  Binding protein for cyclosporin A  Participates in substrate maturation  Participates in substrate maturation  Participates in substrate maturation  Participates in substrate maturation  Participates in substrate maturation  Participates in substrate maturation  Participates in substrate maturation  Participates in substrate maturation  Participates in substrate maturation  Participates in substrate maturation  Participates in substrate maturation |
