# Supplementary material for: Chaperone expression profiles correlate with distinct physiological states of Plasmodium falciparum in malaria patients
Source: Malar J. 2010 Aug 19;9:236. doi: 10.1186/1475-2875-9-236 (PMC2933700; doi:10.1186/1475-2875-9-236)
Supplement: Additional file 9 — List of cluster-wise distribution of Hsp40 class of chaperones. Showing cluster wise distribution of Hsp40 class of chaperones. (+) represents the presence and (-) represents the absence of chaperone in particular cluster. The proteins indicated in bold are PEXEL-containing Hsp40s. [file 1475-2875-9-236-S9.DOC]

**Additional file 9. Cluster-wise distribution of Hsp40s**

| **Protein** | **Cluster1** | **Cluster2** | **Cluster3** | **Hsp40 Type** |
| --- | --- | --- | --- | --- |
| **PFA0675w**  **PFL0055c**  **PFA0110w**  **PF11_0509**  **PF11_0512**  **PF11_0513**  PFI0855w  PFE1170w  PF10_0032  MAL13P1.277  MAL13P1.162  PF11_0433  **PF14_0013**  **PFB0085c**  PFL0565w  **PFB0920w**  PF13_0102  PFL0815w  PF14_0359  PFE0135w  PF11_0273  **PF10_0381**  PF10_0058  PF08_0015  **PF11_0034**  PFI0985c  MAL8P1.25  PFE1170w | +  +  -  -  -  -  +  +  +  +  +  -  -  -  -  +  -  -  -  -  +  +  -  -  +  +  +  + | -  -  +  +  +  ­+  -  -  -  -  -  +  +  +  +  +  +  +  +  +  -  -  +  +  -  -  -  - | -  +  -  -  (3b)  (3b)  (3a)  -  -  -  -  -  (3b)  (3b)  -  +  -  -  -  -  -  +  -  -  +  -  -  - | IV  III  IV  IV  IV  III  -  III  III  II  -  -  IV  IV  II  III  III  III  I  III  III  IV  III  -  IV  III  -  III |
